# Transition of deformation modes from bending to auxetic compression in origami-based metamaterials for head protection from impact

**DOI:** 10.1038/s41598-023-39200-8

**Published:** 2023-07-27

**Authors:** Sunao Tomita, Kento Shimanuki, Shin Oyama, Hidekazu Nishigaki, Toshiaki Nakagawa, Masakazu Tsutsui, Youhei Emura, Masahiko Chino, Hirokazu Tanaka, Yoshinobu Itou, Kazuhiko Umemoto

**Affiliations:** 1 Toyota Central R&D Labs., Inc., Nagakute, 480-1192 Japan; 2 Vehicle Structure & Performance Development Division, TOYOTA AUTO BODY CO.,LTD., Kariya, 448-8666 Japan; 3 Vehicle Architecture Engineering Division, TOYOTA AUTO BODY CO., LTD., Kariya, 448-8666 Japan

**Keywords:** Mechanical engineering, Mechanical properties

## Abstract

For the protection of the human head by energy absorption structures, a soft mechanical response upon contact with the head is required to mitigate the effect of impact, while a hard mechanical response for highly efficient energy absorption is required to stop the movement of the head. This study realized the opposite mechanical properties during head protection by transitioning the deformation mode from bending to auxetic compression. First, non-linear finite element (FE) models were constructed to numerically reproduce the bending behavior. The calculated force responses agreed well with forces in bending tests. Using the FE models, the EA structures with proper transition of deformation modes were designed and installed in the seat headrests of real vehicles. Head protection was evaluated by dynamic loading in sled testing, in which the force on the head of the crash test dummy was measured. The head injury criterion improved from 274 to 155, indicating the superior performance of the tested structures compared to that achieved by energy absorption structures based on steel plates. Moreover, the deformation of auxetic structures prevented neck bending by holding the head. These findings present new possibilities for effectively protecting the human body by mitigating impact, facilitating energy absorption, and ensuring head stability.

## Introduction

Mechanical metamaterials have attracted considerable attention owing to their unique mechanical properties^1,2^. Among mechanical metamaterials, structures with negative Poisson’s ratios have been considered potential candidates for use as auxetic structures^3^. Compared to typical materials expanding under compression, auxetic structures contract laterally. This auxeticity improves the indentation resistance at points where concentrated loads are applied^4,5^, resistance against shear deformation^6,7^, and fracture toughness^8^. Furthermore, they improve the efficiency of energy absorption (EA)^9^. In addition, out-of-bending of auxetic structures generates curved surfaces with positive Gaussian curvatures^10^. For the application of auxetic structures, the mechanical properties and deformation modes of auxetic structures have been extensively investigated^11^. Examples include re-entrant honeycombs^9,12–15^, chiral structures^16,17^, arrowheads^18,19^, inverted tetrapods^20^ and rigid rotation^21,22^. More recently, in addition to auxetic structures, metamaterials with zero Poisson’s ratio have been applied for energy absorption^23,24^.

Moreover, origami-based structures play an important role in the efficient design and fabrication of auxetic structures due to their unique mechanical properties, including auxeticity, and their potential for efficient fabrication. Notable examples of origami-based structures exhibiting auxetic behavior include Miura-ori^25–28^ and water bomb tubes^29–31^. In addition, Tachi–Miura polyhedra (TMP)^32–34^ generate auxeticity owing to their re-entrant shapes obtained by folding^35,36^, and their EA has been investigated^37^. In addition, multidirectional auxeticity based on Miura-ori^38^ and functionally graded auxetic structures based on Miura-ori^39^ were investigated. The main advantages of origami-inspired structures in realizing auxetic structures lie in their efficient fabrication and reconfigurability of mechanical properties. In the context of fabrication, origami-based structures offer potential applications in the creation of cellular structures through plate folding^40^ or self-folding 4D printing techniques^41,42^. In addition, reconfigurability of mechanical properties such as load-bearing capability can switch the performance of EA via folding motion, enabling adaptation to various types of collision scenarios.

In addition to energy absorption from the compression of the auxetic structures, bending deformation has been explored. Sandwich structure bending with re-entrant honeycombs^43–45^, sandwich structures with a star-triangular auxetic honeycomb^46^, and tube structures with a re-entrant honeycomb filler^47^ were investigated via quasi-static testing. In addition, the bending behavior of sandwich structures with re-entrant cores under dynamic loading has also been investigated^48,49^. Moreover, the bending of auxetic structures generates positive Gaussian curvatures. Therefore, curvatures were designed by optimizing the auxetic structures^50–53^.

Because the bending of a conventional plate is a common approach for EA structures^54^, the bending of an auxetic structure can lead to various beneficial mechanical properties and deformation modes. However, although conventional studies have considered that the positive Gaussian curvatures by bending of auxetic structures improve comfort by conforming to human bodies^55^, to the best of our knowledge, previous research has not investigated the bending behavior of auxetic structures specifically for human protection. In the case of head protection using energy absorption structures, it is necessary to have a soft mechanical response upon contact with the head to mitigate the impact effect, while a hard mechanical response is required to efficiently absorb energy and prevent head movement. Therefore, this study proposes auxetic structures for human protection that transition deformation modes from bending to auxetic compression to provide both soft and hard mechanical properties. The proposed structure has the potential to offer the following benefits for human safety: the transition of deformation modes allows for a gradual shift from a soft to a hard force response, achieving impact mitigation after contact and efficient energy absorption to minimize body movement. Moreover, the curvature caused by the bending of auxetic structures can enhance the performance of holding the human body, which decreases the injury caused by the movement of the human body and the second impact.

**Figure 1:**
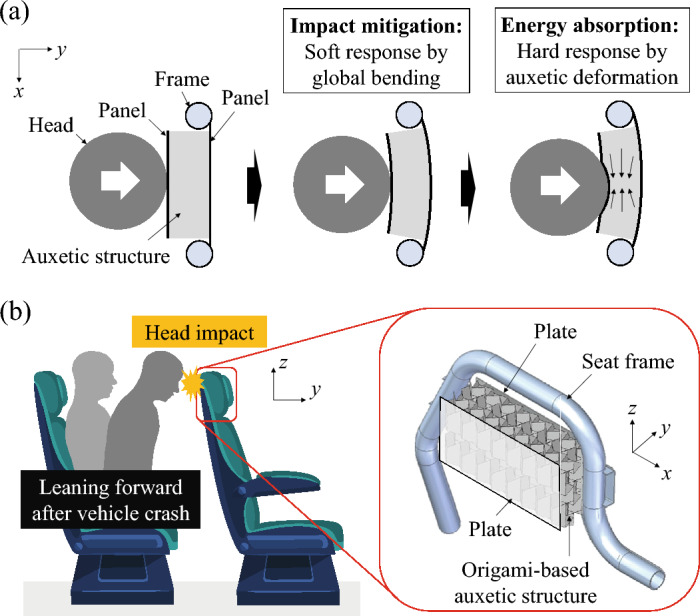
Realization of both impact mitigation and the EA based on the transition of deformation modes. (**a**) Schematic of the deformation-mode transition for impact mitigation and EA. The bending mode provides a soft response for impact mitigation and auxetic deformation provides a hard response for EA. (**b**) The specific application for head protection from the collision between the head and headrest of a seat in a vehicle.

This study demonstrated head protection based on the transition of deformation modes from bending to auxetic compression, as shown in Fig. 1a. The transition of the deformation mode provides soft mechanical responses for impact mitigation after contact with the head and hard mechanical responses for high-efficiency EA during the deformation of auxetic structures. This concept was verified through experimental and numerical quasi-static testing to eliminate the oscillation by impulse responses in dynamic testing. In a quasi-static compression test, the dimensions and shape of the test pieces were designed to achieve the desired rigidity, load capacity, and collapse length. Furthermore, to evaluate the performances of EA structures against collision, it is important to consider the effect of dynamic loading because the strain rate dependency and breaking by impact loading change the mechanical responses of EA structures^56–58^. Therefore, the proposed structure was applied to an important situation that required impact mitigation: the collision of a human body against a seat during a vehicle crash^59–62^. The situation, which is regulated by UN-R80^63^, was tested as shown in Fig. 1b. In addition, the head injury criterion (HIC)^64^, which is typically used as an indicator for injury in crash tests, was compared with the HIC obtained from tests involving conventional EA plate structures.

## Design

**Figure 2:**
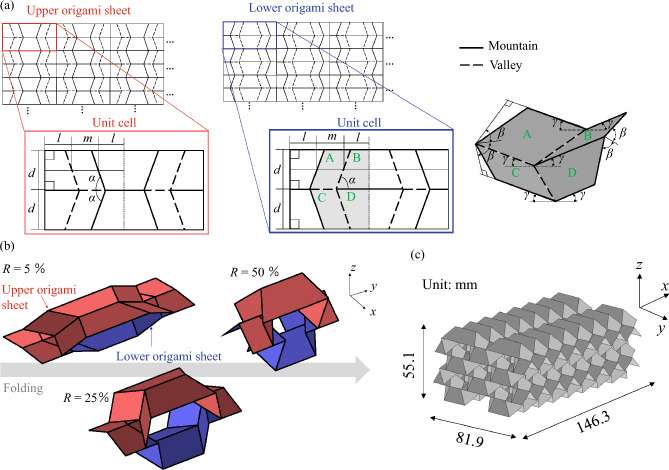
Geometry of TMP. (**a**) Crease patterns of upper and lower origami sheets and the folding motion around the vertex. (**b**) Geometrical change of TMP comprises the upper and lower origami sheets ($l:m:d = 1:1:1, \alpha = 65^\circ$). (**c**) Geometry of the auxetic structure was designed by the tessellation of $8\times 2\times 2$-unit cells of the TMP.

The TMP unit cell was created by pairing the upper and lower origami sheets, as shown in Fig. 2a. The geometry is defined by the lengths *l*, *m*, and *d* and the internal angle of the parallelogram $\alpha$. The change in the TMP geometry is characterized by the folding angles $\beta$, $\gamma$ and the internal angle of the parallelogram $\alpha$^32^:

1 $$\begin{aligned} \tan (\gamma )=\tan (\alpha )\cos (\beta ). \end{aligned}$$

To show the state of the TMP, the folding ratio related to $\beta$ was defined as follows:

2 $$\begin{aligned} R=\frac{90^\circ -\beta }{90^\circ } \times 100 [\%]. \end{aligned}$$

The cross sections of TMP tubes changed from convex shapes (for example, $R=25\ \%$ in Fig. 2b) to concave shapes (e.g. $R=50\ \%$ in Fig. 2b) as the folding ratio increased, where TMPs were created by pairing upper and lower origami sheets with $l: m: d = 1:1:1$, $\alpha = 65^\circ$. Because of the cross-sectional change, re-entrant shapes occur in TMP, which leads to auxeticity.

The auxetic structures were designed and fabricated based on TMPs. As an example of auxetic structures with highly efficient EA, the geometrical parameters $l = m = d = 12.5\ \textrm{mm}$, $\alpha = 65^\circ$ and $R =47.8\ [\%]$ were employed by scaling the TMPs used in a previous study^37^. These parameters provide auxeticity caused by the re-entrant shapes in the TMP. To install the auxetic structures in the headrest used in commercial vehicles, the tessellation of $8\times 2\times 2$-unit cells shown in Fig. 2c provides an auxetic structure with dimensions of $146.3\ {\textrm{mm}}\ (x)\ \times 81.9\ {\textrm{mm}}\ (y) \times 55.1\ {\textrm{mm}}\ (z)$, which readily fits in real vehicle headrests. The number of tessellations were determined by confirming that the auxetic behavior is maintained during compression.

## Manufacturing

**Figure 3:**
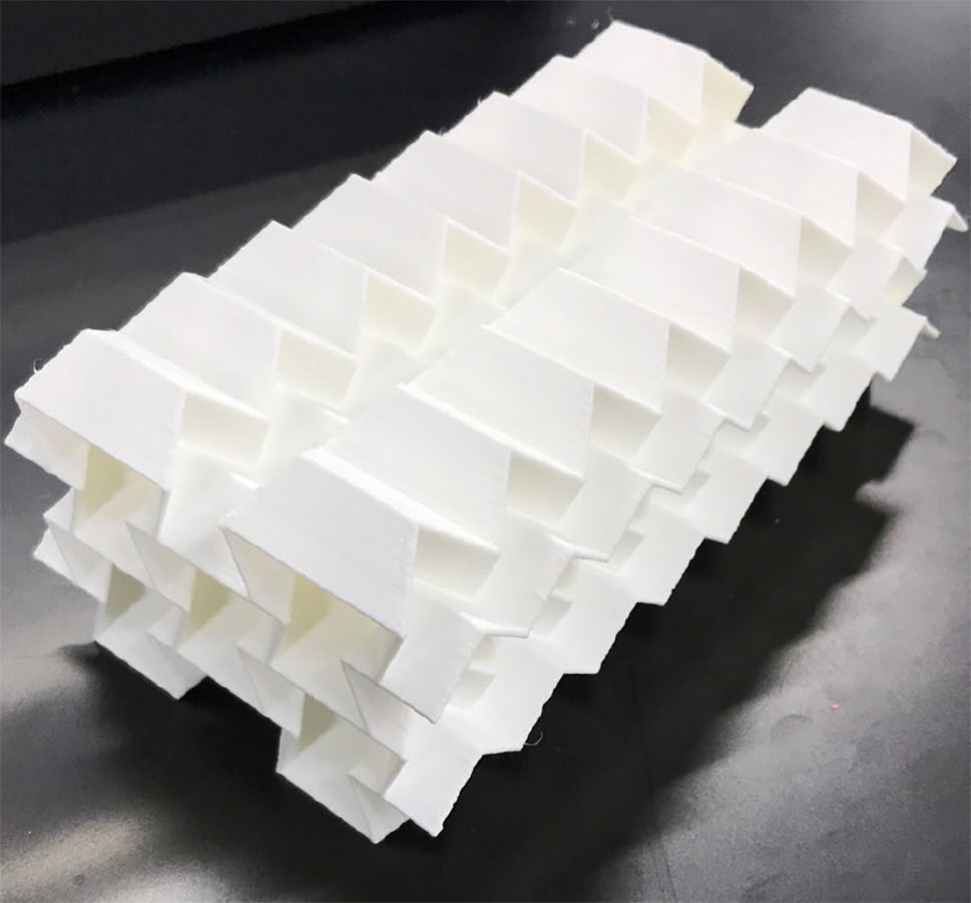
Fabricated auxetic structure designed by the tessellation of $8\times 2\times 2$-unit cells of the TMP.

The designed TMPs were fabricated as shown in Fig. 3. A fused filament fabrication (FFF)-type 3D printer (Flashforge, Adventurer3X) and commercially available nylon filament with a diameter of 1.75 mm (Markforged, Nylon White) were used to fabricate the auxetic structures. The test pieces were stored in water at 50 $^\circ \hbox {C}$ for 20 h before the experiments to ensure that the nylon was in a water-absorbed state. This procedure was adopted because water absorption causes the nylon state to transition from glassy to rubbery.

## Experimental setup/test

### Quasi-static testing

**Figure 4:**
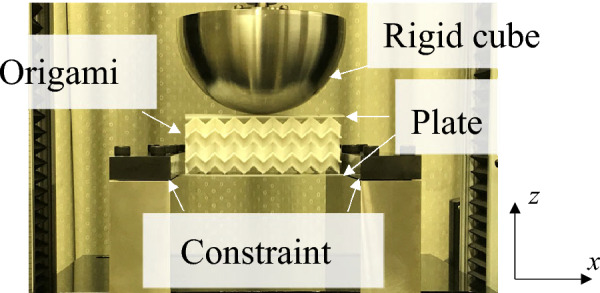
Experimental setup of quasi-static bending test of sandwich structures with auxetic-cores. (compression speed was 5 mm/min).

To experimentally verify the deformation mode transition, the force response of auxetic samples sandwiched between upper and lower plates was measured through quasi-static bending tests, as shown in Fig. 4. The sandwich structures were constrained at the left and right boundaries of the lower plates. The upper and lower panels were 1-mm-thick nylon and aluminum (A5052), respectively. The auxetic structures were compressed by moving a cube whose diameter was 165 mm downwards.

A standard compression-testing machine (Instron 5566) was used to perform quasi-static bending tests. The maximum deformation was 50 mm, and the compression speed was set to 5 mm/min to prevent inertial effects. The compression of the test pieces was recorded using a digital video camera (Panasonic HC-W870M).

### Dynamic testing

**Figure 5:**
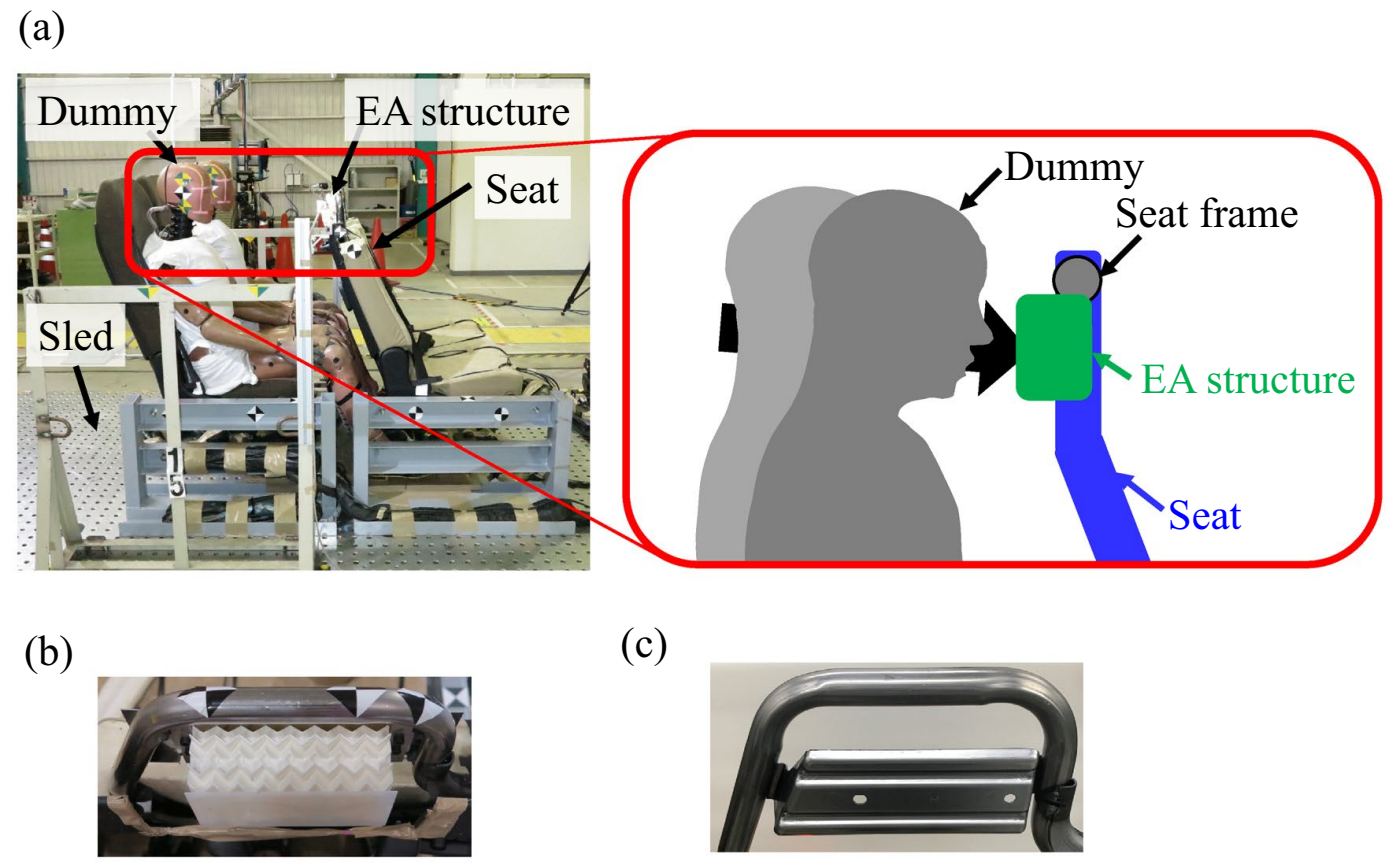
Experimental setup for sled testing. (**a**) The equipped sled and seat are accelerated by compressed air based on UN regulations. The crash-test dummies set on the seats move frontward, and their heads collide with the EA structures set in the seat. (**b**) Auxetic structures in the seat frame and (**c**) steel plates commonly used EA structures.

To imitate the dynamic load caused by the acceleration experienced during a vehicle crash, a commercial sled test system driven by compressed air was used, as shown in Fig. 5a. The sled testing was conducted using 1.2-mm-thick auxetic structures. The lower panels were made of two 1-mm-thick aluminum plates layered together and connected to the seat frame using bolts. In addition, the top of the auxetic structures was covered with a 1-mm-thick nylon plate, as shown in Fig. 5b. A commercial 1.4-mm-thick cold steel plate (JIS G 3141) with trapezoidal shapes (upper dimension of 160 mm, lower dimension of 200 mm, and height of 75 mm) was also used in the sled testing, as shown in Fig. 5c, to contrast the performance of the auxetic plates against common EA structures.

The acceleration was generated based on the UN-80 regulation^63^ using compressed air to move the commercial sled test system (HyperG 220, Dr. Steffan Datentechnik). The forces on the head, chest, and neck of the dummy set on the right seat (front side of Fig. 5a) were measured during the test, and the movement of the head was recorded via the sled test system. The HIC was calculated as^64^:

3 $$\begin{aligned} {\textrm{HIC}}={\textrm{max}}\left( {t_2}-{t_1}\right) \left( \left\{ \frac{1}{{t_2}-{t_1}}\right\} \int ^{t_2}_{t_1}a(t)dt\right) ^{2.5}, \end{aligned}$$

where *a* is acceleration at head of dummy, $t_1$ and $t_2$ are two time points such that $0<{t_1}<{t_2}<T$, and *T* is the duration of the impact.

## Finite element analysis (FEA)

**Figure 6:**
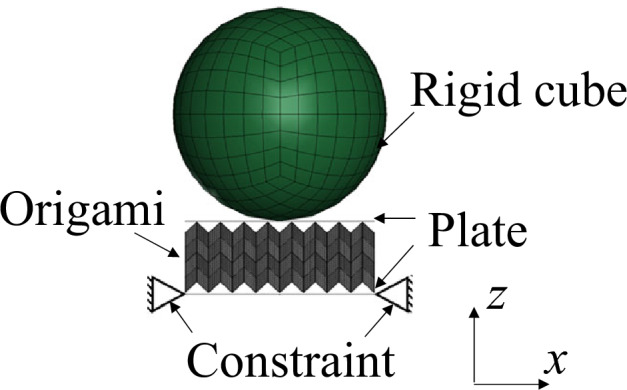
Boundary conditions for finite element analysis.

To numerically reproduce the deformation process during the bending experiments, nonlinear finite element analysis (FEA) was performed using a commercial software (LS-DYNA^65^), as shown in Fig. 6. A quasi-static simulation was performed using an explicit dynamic scheme to consider the contact between the auxetic panels under a stable state. The auxetic structures were constrained at both the left and right edges of the lower plates of the sandwich structures. The auxetic structures were deformed by the downward displacement of a rigid cube. Four-node shell elements were used to model the panels. Furthermore, the materials were assigned elastoplastic constitutive laws based on the stress–strain curves of the auxetic cores and upper panels, which were obtained from three-point bending tests performed on rectangular plates additively manufactured with the same materials used in the fabrication of the auxetic structures. The Young’s modulus obtained from the three-point bending test was 332 MPa. The mass density and Poisson’s ratio were 1162 ${\mathrm{{kg}}/{\text {m}}}^3$ and 0.4, respectively, as shown in Table 1. In addition, the lower plates were modeled using the stress–strain curve of A5052^66^, with Young’s modulus, density, and Poisson’s ratio of 70 GPa, 2910 $\textrm{kg}/\textrm{m}^3$, and 0.33, respectively^67^. The thickness of the shell elements adjacent to the crease of the origami was increased by 1 mm. The friction between the panels was set as 0.2, and that between the panels and the rigid cube was set to 0.5.

**Table 1:** Properties of nylon materials.

Young’s modulus	Poisson’s ratio	Mass density	Breaking strain
332 [MPa]	0.4	1162 [$\textrm{kg}/\textrm{m}^3$]	0.95

## FEA validation

**Figure 7:**
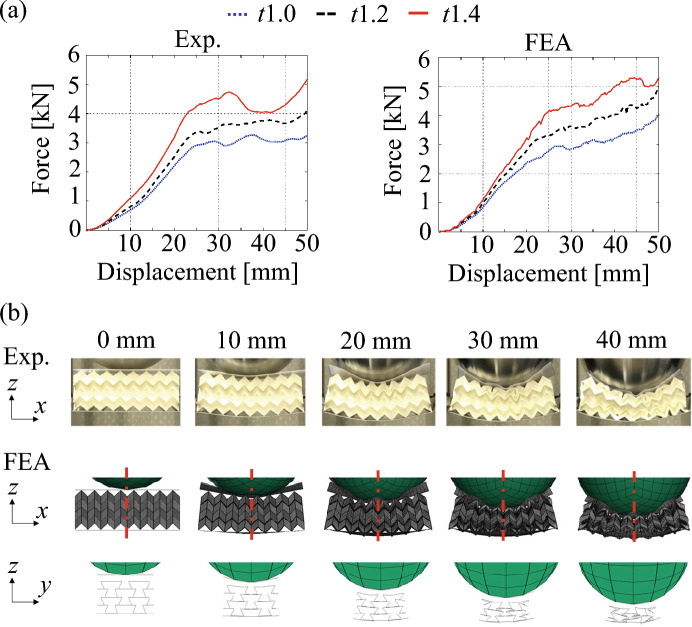
Quasi-static bending test of sandwich structures with auxetic-cores. (**a**) Comparison of force-displacement curves between the thickness of TMPs. (**b**) Deformation of the auxetic structures by contact with rigid cubes in experimental and numerical testing.

Figure 7a shows the force-displacement curves during compression of the auxetic structures sandwiched with panels by rigid cubes through experiment and FEA. The tests were performed for auxetic structures with 1.0-, 1.2-, and 1.4-mm-thick panels. The force responses increased linearly with the displacement of the rigid cube until approximately 20-mm deformation, where global bending is dominant (i.e., the re-entrant shapes of TMPs in *zy*-plane do not shrink in FEA.) as shown in Fig. 7b. However, the force responses plateaued after the deformation mode transitioned to auxetic compression (i.e., the re-entrant shapes of TMPs shrank in FEA under 30- and 40-mm deformation). Therefore, the proposed structures exhibit soft mechanical properties under small deformations and hard mechanical properties under large deformations. The transition of mechanical properties is suitable for the realization of both impact mitigation after impact and high EA to prevent head movement. In addition, the plateau force owing to auxetic compression changed with the thickness of the plates consisting of auxetic structures, which enabled us to easily adjust the EA.

**Figure 8:**
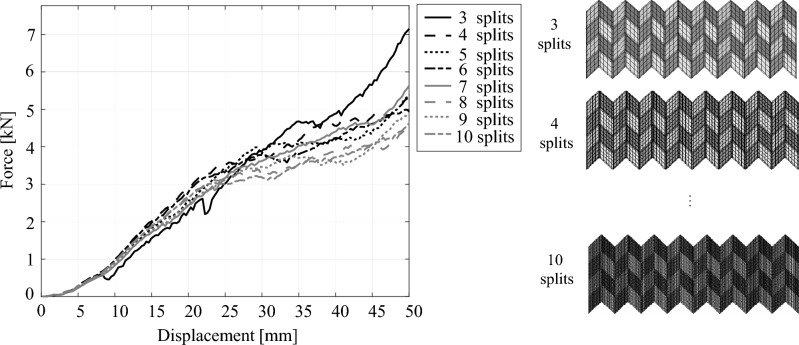
Effects of the mesh size on force responses. In addition to the FE model discretized by mesh size with three splits of the side of the parallelogram, the force responses are stable.

Furthermore, the effects of mesh sizes were investigated by changing the number of splits in the side of parallelogram from 3 to 10 as shown in Fig. 8. Although force responses obtained from FE model discretized with mesh by three splits were unstable, other force responses were approximately stable among the FE models discretized by different mesh sizes. In this study, the FE models were discretized with mesh sizes with six splits, which is sufficient to obtain stable force responses.

## Results and discussion

### Investigation of design parameters

**Figure 9:**
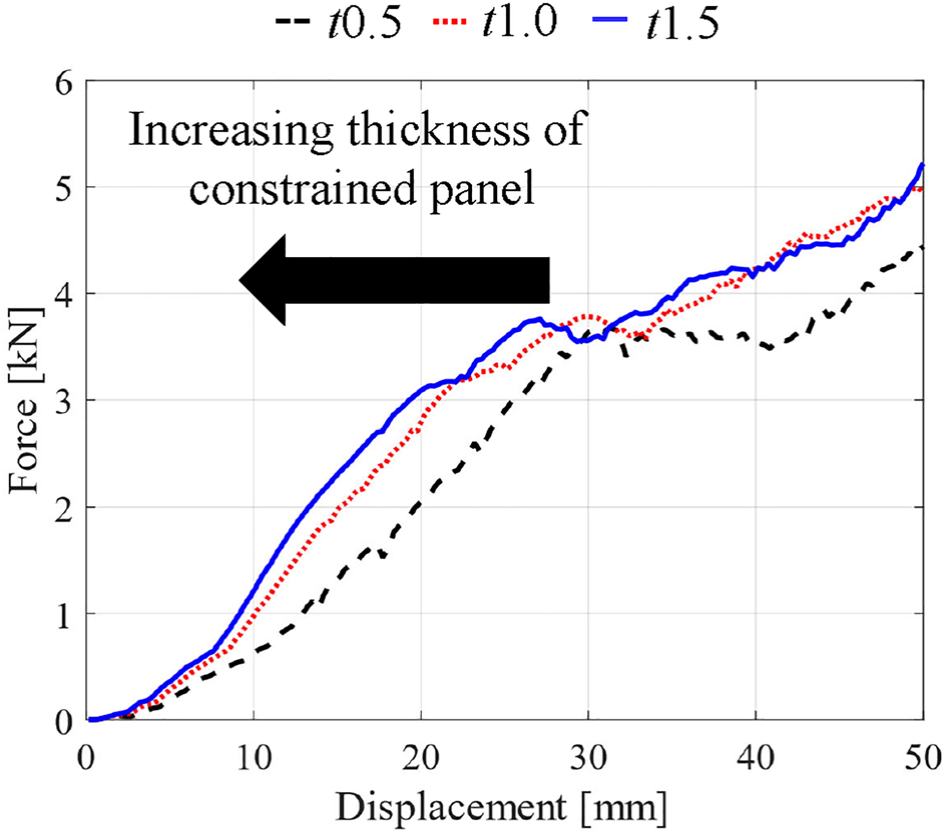
Effects of the thickness of constrained panels on global bending deformation.

To design the bending deformation of the EA structures, the effects of the thickness of constrained plates were investigated by changing the thickness of the constrained panels from 0.5 mm to 1.5 mm in FEA used in Fig. 7. As shown in Fig. 9, the larger thickness of constrained panels increases the slope between displacement and force, which transits deformation modes from bending to auxetic compression in small displacements. In contrast to the difference in global bending, the thickness of constrained panels has no significant influence on the plateaued force. Therefore, we can independently design soft responses for impact mitigation by considering the thickness of the constrained plate.

**Figure 10:**
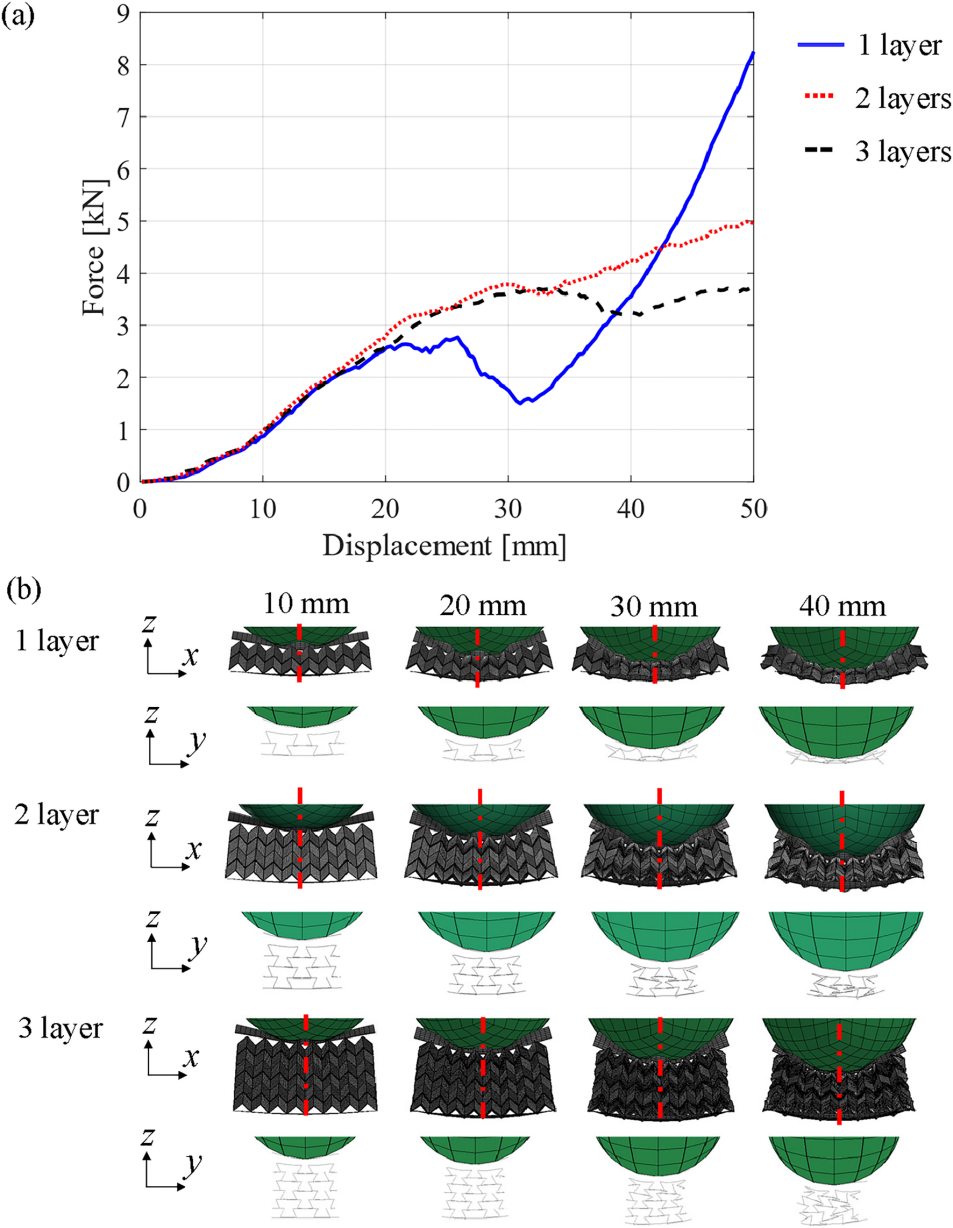
Comparison of quasi-static responses between 1-, 2- and 3-layered TMPs. (**a**) Comparison of force-displacement curves (**b**) Deformation of the auxetic structures by contact with rigid cubes.

Furthermore, the numbers of stacked layers were investigated by changing the number of tessellation of the TMPs in the *z*-direction based on the FEA models used in Fig. 7. As shown in Fig. 10a, the force response of the 1-layered TMP plateaued at approximately 20-mm displacement. However, the lack of TMP cells disrupted the auxetic behavior (i.e., the cross-section in *zy*-plane expands during deformation) as shown in Fig. 10b, which caused a dip of force responses in approximately 30-mm displacement and increased the force by densification for displacements larger than 40 mm. In contrast, as the auxetic behavior of the 2-layered TMP was not broken (i.e., the cross-section in *zy*-plane shrinks during deformation), the plateaued force response continued in the long displacement region. Moreover, the forces of 3-layered TMPs were more ideally plateaued. These results suggest that a larger number of layers in TMP provide plateaued forces in the long displacement region. In contrast to the plateaued phase, the global bending in the early stage of displacement was not influenced by the number of layers because TMPs flexibly deform in *x*-axis direction by their one-degree-of-freedom motions. Therefore, we can choose the number of layers to satisfy the required EA under geometric limitations. In this study, the 2-layer TMPs were installed in the headrests as the minimum number of layers for obtaining the long plateaued force.

### Energy absorption under dynamic loading

**Figure 11:**
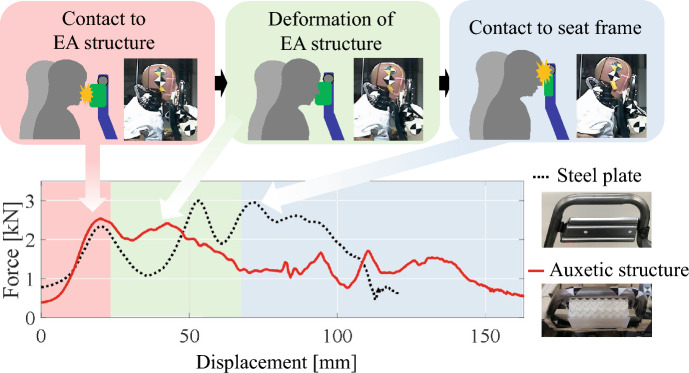
Force at the head of the dummy during the sled test. After the contact between the head and EA structures, the head is subjected to a force of approximately 2.0–2.5 kN and the auxetic structures provide high EA due to their plateau force response, preventing the head from making contact with the seat frame.

Figure. 11 shows the comparison of force responses on the head from steel plate and auxetic structures. Because the steel plate causes a dip in the force response after contact with the head (20-mm displacement), the lack of EA generates force peaks in the later stage of deformation by the contact of the head with the seat frame (75-mm displacement). In contrast, the auxetic structures generate a plateau force response (from 20 to 40-mm displacement) after contact with the head without increasing the initial peak of the force responses compared to the steel plates, which provides a highly efficient EA, as confirmed in the quasi-static testing shown in Fig. 7. Owing to the difference in EA, the contact between the head and seat frame that occurred around a 75-mm displacement in the steel plate was prevented by the auxetic structure; consequently, the maximum force on the head reduced from 3 to 2.5 kN. Furthermore, the HIC associated with the likelihood of head injury significantly improved from 274 to 155. Therefore, the proposed concept provides efficient head protection. Although the auxetic structures were designed via quasi-static loading, the low initial peaks and plateaued force responses were maintained in the dynamic loading by sled testing. Therefore, the proposed structures effectively mitigated high-velocity impacts, such as those encountered in vehicle crashes.

**Figure 12:**
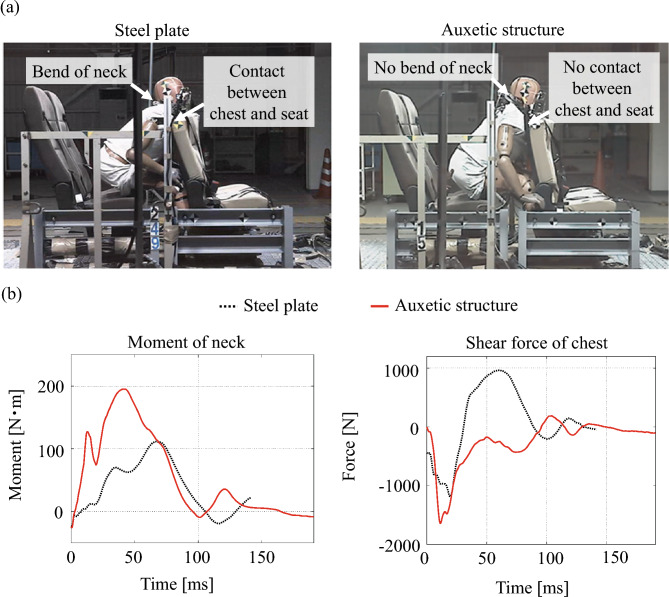
Comparison of dummy motions. (**a**) The motion of the dummy. (**b**) The moment of the neck and shear force of the chest.

Furthermore, the motions of the crash test dummy were compared between sled tests using a steel plate and the auxetic structures, as shown in Fig. 12a. The neck of the dummy bent during the deformation of the steel plate, resulting in contact between the chest and seat, as shown in Fig. 12a. However, the neck of the dummy does not bend after contact with the auxetic structure, which prevents contact between the chest and seat. The difference in motion is evidenced by the moment of the neck and the shear force of the chest, as shown in Fig. 12b. The prevention of neck bending relies on the unique curvature caused by the bending of the auxetic structures. Although conventional plates (i.e., those with a positive Poisson ratio) generate negative-Gaussian curvatures (i.e., hyperbolic surfaces), it is known that the bending of auxetic structures generates positive-Gaussian curvatures (i.e., bowl-shaped). The bowl curvature can easily fit the shape of the head. In contrast, because the bending of a conventional steel plate does not generate a curvature fitting to the head, the head of the dummy appears to slip upward after contact. Therefore, the bending of auxetic structures could have the potential to improve head protection by deformation, allowing easy fitting around the head.

## Conclusion

This study demonstrated head protection in a vehicle crash situation based on the transition of the deformation mode from bending to auxetic compression, which provides soft mechanical responses for impact mitigation after contact with the head and hard mechanical responses for high-efficiency EA during the deformation of auxetic structures. First, the transition of the deformation mode was modeled by FE analysis, and the models were verified by experimental quasi-static bending tests. Following the deformation mode, their force responses also shifted from soft to hard and plateaued. Furthermore, the auxetic structures were equipped on the headrests of seats in real vehicles, and head protection was evaluated by sled testing. The force on the head of the crash test dummy from the headrest was evaluated under artificial acceleration conditions imitating a vehicle crash. In the sled testing, the maximum force at the head reduced from 3 to 2.5 kN, and the HIC significantly improved from 274 to 155 compared to the case of common steel-plate EA structures. Furthermore, the deformation of auxetic structures prevented the bending of the neck by holding the head. Therefore, the transition of deformation modes in auxetic structures is beneficial for the efficient protection of the human body via the realization of impact mitigation, EA, and head holding.

In this study, the condition of dynamic loading was limited to head collision under vehicle crash situations. To extend the application of the proposed structures to various types of crash scenarios, it is important to investigate the effect of strain rate in wider applications. Furthermore, it is important to explore the bending behavior of various types of auxetic structures, in addition to Tachi–Miura polyhedron, for investigating the effect of geometries of auxetic structures on the EA and human protection.

## Data Availability

The datasets generated and analyzed during the current study are available from the corresponding author upon reasonable request.
